# Synthesis, Spectroscopic Characterization, X-Ray Structure, and DFT Calculations of Some New 1,4-Dihydro-2,6-Dimethyl-3,5 -Pyridinedicarboxamides

**DOI:** 10.1371/journal.pone.0091361

**Published:** 2014-03-13

**Authors:** Yi Li, Yuan-Yuan Liu, Xue-Jun Chen, Xiao-Hui Xiong, Fang-Shi Li

**Affiliations:** 1 College of Food Science and Light Industry, Nanjing Tech University, Nanjing, China; 2 Department of Chemical and Pharmaceutical Engineering, Southeast University ChengXian College, Nanjing, China; 3 College of Science, Nanjing Tech University, Nanjing, China; University of Calgary, Canada

## Abstract

A series of novel 1,4-dihydro-2,6- dimethyl-3,5-pyridinedicarboxamides were synthesized and characterized by infrared absorption spectrum (IR), proton nuclear magnetic resonance (^1^H NMR), elemental analysis, ultraviolet spectrum (UV), and fluorescence techniques, together with X-ray single crystal diffraction. The results of density functional theory (DFT) and time-dependent density functional theory (TDDFT) calculations provided a reasonable explanation on the molecular structures, the molecular frontier orbital, and the spectra of electronic absorption and emission. The present work will be helpful to systematically understanding of the structures and the optical properties of 1,4-dihydropyridines for studying the structure-activity relationship and to develop new drugs and their analytical methods.

## Introduction

1,4-Dihydropyridines (1,4-DHPs) are very important bioactive molecules in the field of drug and pharmaceuticals. These compounds are well known as calcium channel modulators and have emerged as one of the most important classes of drugs for the treatment of hypertension and so on [1]–[3]. Owing to the potential importance of 1,4-DHPs from pharmaceutical, industrial and synthetic points of view, the synthesis of 1,4-DHPs has attracted much attention and various methods have been developed [4]. Hantzsch reaction is the most classical method for the synthesis of 1,4-DHPs, by which a mixture of *β*-keto ester, an ammonium salt and an aldehyde in organic solvents is heated together [5]–[7]. It has been demonstrated that substitution of aryl-amide group for dicarboxylic esters moiety reduces the Ca^2+^ channel blocker activity and increases antitubercular activity [8]–[10].

To the best of our knowledge, neither the crystal structure nor the theoretical studies of spectroscopy for such compounds have been reported up to now. This inadequacy observed in the literature encouraged us to do this research based on experimental techniques and theoretical method.

**Figure 1:**
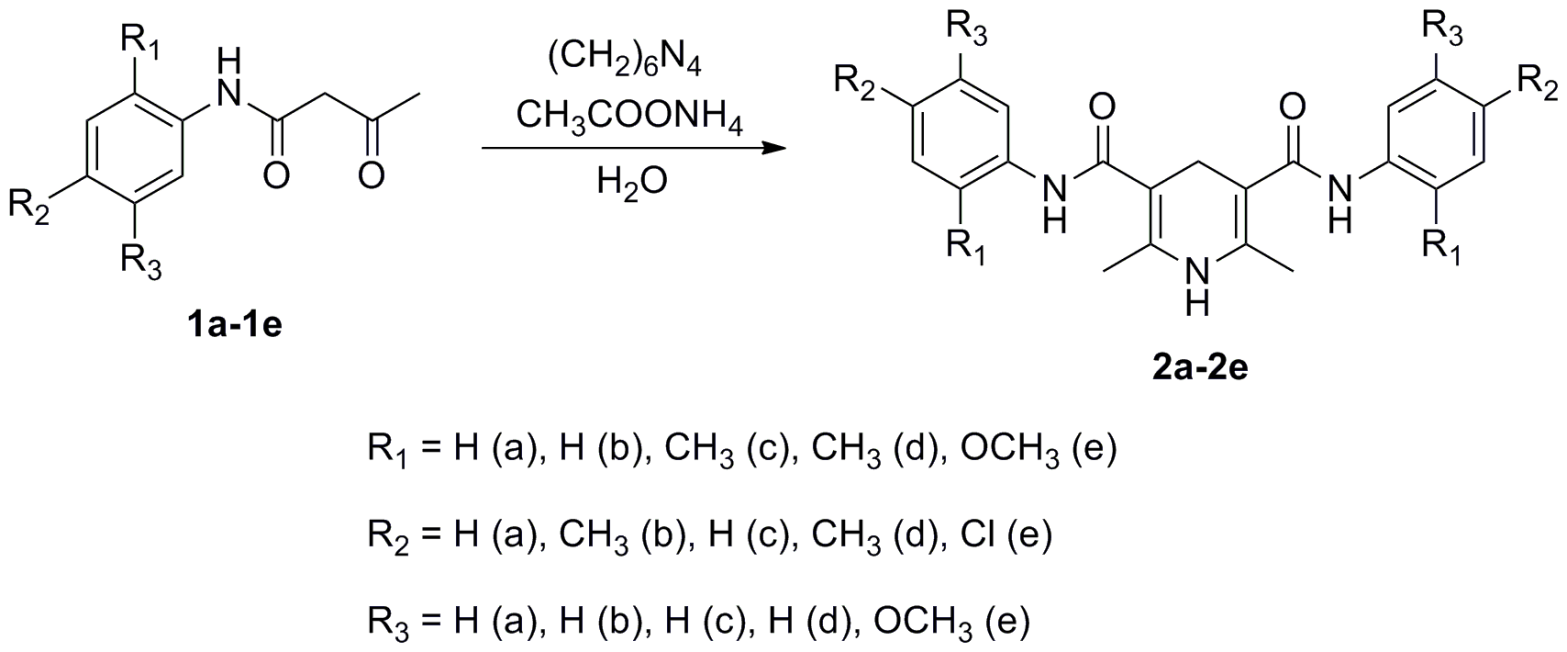
Synthetic routes of compounds a–e.

In this work, five novel 1,4-dihydro-2,6-dimethyl-3,5-pyridinedicarboxamides (**a-e**) with different substituents on the benzene rings were synthesized (**Fig. 1**). The structures of **a–e** were characterized by IR, ^1^H NMR, elemental analysis, UV-Vis, fluorimetry, and single-crystal X-ray diffraction. The structures, frontier orbital, and optical properties of the compounds were investigated by using density functional theory (DFT) and time-dependent density functional theory (TDDFT) to provide theoretical understanding.

## Results and Discussion

### Description of the crystal structures

**Figure 2:**
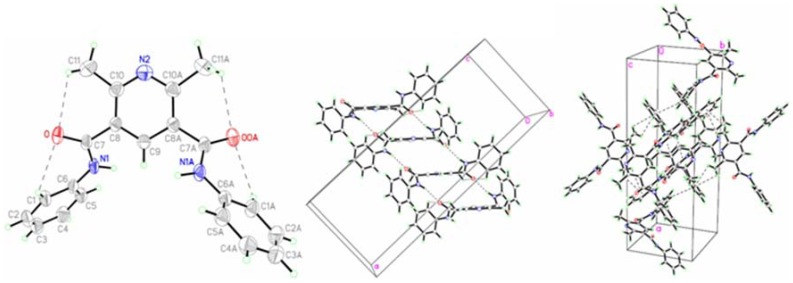
Crystal structure of a.

**Figure 3:**
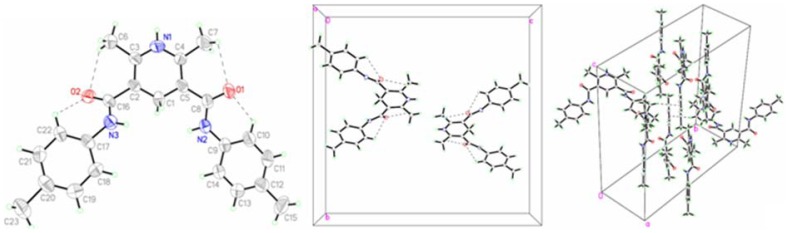
Crystal structure of b.

The crystals of **a** and **b** were prepared and determined by single crystal X-ray diffraction. Their crystal data and structure refinement are shown in **Table 1**. The selected bond lengths and angles are tabulated in **Table 2**. The observed hydrogen bonds are listed in **Table 3**. The molecular ellipsoid and the unit cell accumulation are shown in **Figs 2** and **3**, respectively.

**Table 1:** Crystal data and structure refinement for a and b.

	a	b
CCDC No.	851885	851886
empirical formula	C_21_H_19_N_3_O	C_23_H_25_N_3_O_2_
formula weight	345.40	375.46
temperature [K]	293(2)	293(2)
wavelength [Å]	0.71073	0.71073
crystal system,	Orthorhombic	Orthorhombic
space group	*Pbcn*	*Pbca*
unit cell dimensions		
*a* [Å]	23.278(5)	8.5660(17)
*b* [Å]	8.3400(17)	21.824(4)
*c* [Å]	9.4260(19)	22.631(5)
*α* [°]	90.00	90.00
*β* [°]	90.00	90.00
*γ* [°]	90.00	90.00
volume [Å^3^]	1829.9(6)	4230.7(15)
*Z*	4	8
*ρ*_calcd_ [g cm^−3^]	1.254	1.179
μ [mm^−1^]	0.075	0.076
*F*(000)	728	1600
crystal size [mm^3^]	0.10 × 0.20 × 0.30	0.10 × 0.10 × 0.20
*θ* range [°] for data collection	1.75 to 25.37	1.80 to 25.37
index ranges	−28≤h≤28	0≤h≤10
	0≤k≤10	0≤k≤26
	0≤l≤11	−2≤l≤27
reflections collected	3304	4308
independent reflections	1676 [*R*_int_ = 0.040]	3879 [*R*_int_ = 0.075]
max. and min. transmission	0.9918/0.9757	0.9924/0.9849
data/restraints/parameters	1676/0/120	3879/8/253
goodness-of-fit on *F* ^2^	1.000	1.001
final *R* indices [*I*>2*σ*(*I*)]; *R*_1_, *wR*_2_	0.0506, 0.1382	0.0772, 0.1140
*R*_1_, *wR*_2_ (all data)	0.0849, 0.1585	0.2084, 0.1488
largest diff. peak and hole [e·Å^−3^]	0.241 and −0.150	0.149 and −0.180

**Table 2:** Selected crystal structure parameters of a and b.

**Parameters**	**a**		
**Bond lengths (Å)**	**Experimental**	**B3LYP/6-31G(d)**	**B3LYP/6-31G(d, p)**
O-C7	1.227(2)	1.2335	1.2337
N1-C7	1.348(3)	1.387	1.3866
N1-C6	1.405(3)	1.4098	1.4093
N2-C10	1.343(3)	1.384	1.3837
**Bond angles (°)**			
C7-N1-C6	127.10(18)	129.1118	129.0683
C10-N2-C10A	120.9(3)	125.4339	125.3796
C5-C6-N1	118.3(2)	116.8468	116.8731
C1-C6-N1	122.7(2)	123.9904	123.9536
O-C7-N1	123.4(2)	121.6942	121.746
O-C7-C8	122.5(2)	123.5201	123.497
N1-C7-C8	114.13(17)	114.7855	114.7569
N2-C10-C8	121.2(2)	119.5133	119.5371
N2-C10-C11	115.3(2)	113.6535	113.7053
**Parameters**	**b**		
**Bond lengths (Å)**	**Experimental**	**B3LYP/6-31G(d)**	**B3LYP/6-31G(d, p)**
N2-C8	1.347(4)	1.3861	1.3856
N2-C9	1.414(4)	1.4103	1.4099
O2-C16	1.239(3)	1.2339	1.2342
C3-N1	1.303(4)	1.3841	1.3838
N3-C16	1.360(4)	1.2339	1.2342
N3-C17	1.436(3)	1.4103	1.4099
N1-C4	1.331(4)	1.3841	1.3838
**Bond angles (°)**			
C8-N2-C9	127.2(3)	129.0404	128.9959
N1-C3-C2	122.5(3)	119.5227	119.5469
N1-C3-C6	115.8(3)	113.6518	113.7043
C16-N3-C17	125.0(3)	129.0401	128.9947
C3-N1-C4	121.4(3)	125.4196	125.3645
N1-C4-C5	121.7(3)	119.5228	119.547
N1-C4-C7	116.7(3)	113.6518	113.7043
O1-C8-N2	123.0(3)	121.6857	121.7401
O1-C8-C5	120.9(3)	123.5064	123.481
N2-C8-C5	116.1(3)	114.8079	114.7788
C10-C9-N2	122.9(3)	124.1974	124.1565
C14-C9-N2	117.8(3)	117.1108	117.1363
O2-C16-N3	121.8(3)	121.686	121.7402
O2-C16-C2	123.5(3)	123.5067	123.4814
N3-C16-C2	114.7(3)	114.8073	114.7784

**Table 3:** Parameters (Å, °) for the intra- and intermolecular interactions in a and b.

Comp.	D-H…A	D-H	H…A	D…A	D-H…A
*(a) Intermolecular and intramolecular hydrogen bond*					
**a**	C1-H1B…O	0.9300	2.4900	2.940(3)	110.00
	C11-H11B…O	0.9600	2.5800	2.987(3)	106.00
	N1-H1A…O^a^	0.8600	2.0700	2.907(2)	166.00
**b**	C6-H6C…O2	0.9600	2.5500	2.958(4)	106.00
	C7-H7C…O1	0.9600	2.5000	2.883(4)	104.00
	C10-H10A…O1	0.9300	2.5000	2.953(5)	110.00
	C22-H22A…O2	0.9300	2.5500	2.940(4)	106.00
Comp.	C-H…*Cg*	C-H	H…*Cg*	C…*Cg*	C-H…*Cg*
*(b) C-H…π interactions*					
**a**	C1-H1B…*Cg*1^b^	0.9300	3.1078	3.942(3)	150.26
	C1-H1B…*Cg*1^c^	0.9300	3.1078	3.942(3)	150.26
	C3-H3A…*Cg*2^d^	0.9300	3.0490	3.832(3)	142.92
	C11-H11A…*Cg*2^e^	0.9600	3.2060	3.567(3)	104.33
	C11-H11C…*Cg*2^e^	0.9600	3.1510	3.567(3)	108.05
**b**	C11-H11A…*Cg*3^f^	0.9300	3.3216	4.212(4)	161.06

Symmetry codes: ^a^x, 1−y, 1/2+z. ^b^1−x,1−y,−z. ^c^x,1−y,−1/2+z. ^d^1/2−x,1/2+y, z. ^e^x,−1+y, z. ^f^1/2−x,−1/2+y, z.

The molecules of **a** and **b** are axial symmetry. The line passing through the N atom and the C atom at the 4-position of pyridine ring is the axis of symmetry of molecules. The amide bond lengths of N1-C7 (1.348(3) Å) in **a** and N2-C8 (1.347(4) Å) in **b** are within normal ranges (1.325–1.352 Å) [11]. The dihedral angel between phenyl ring and pyridine ring is 77.88° in **a** and 6.04° in **b**. It indicates that the phenyl rings are orthogonal to the pyridine ring in **a**, and nearly in co-plane to the pyridine ring in **b**. The dihedral angle of two phenyl rings is 85.19° in **a** and 1.22° in **b**. The intramolecular C-H···O H-bonds in the two molecules result in the formation of four non-planar pseudo six-member rings with envelope conformations. The dihedral angle of the carbonyl and the pyridine ring is 45.02 ° in **a** and 32.37 ° in **b**.

The molecules of **a** are stabilized by intermolecular N-H···O H-bonds and C-H···π stacking interactions, while **b** is organized only by C-H···π stacking interactions. The molecules are interlinked by the intermolecular hydrogen bonds to form an infinite chain.

### Geometric optimization and conformational study

**Figure 4:**
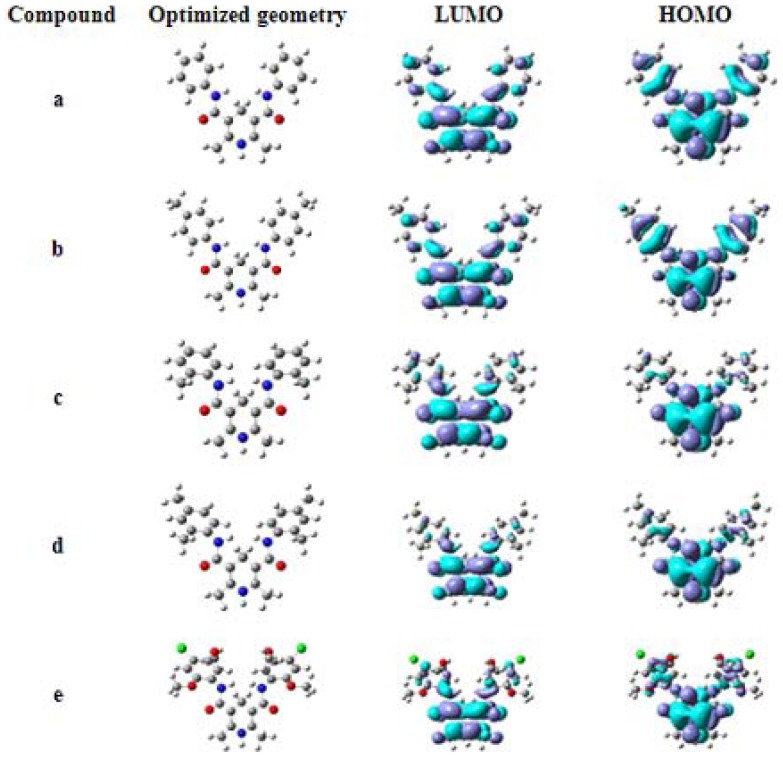
The optimized geometries and the surfaces of the frontier molecular orbital of a-e obtained at the B3LYP/6-31G (d) level.

The structures of **a-e** have been optimized with DFT/B3LYP/6-31G (d). The five molecular conformations look like five different butterflies with beautiful symmetry (**Fig. 4**).

In order to confirm the molecular structures of the compounds, the calculated results of **a** and **b** are presented in **Table 2**, together with the X-ray diffraction data. Because the results of the two calculation methods (6-31G (d) and 6-31G (d, p)) were similar, we used the results calculated by 6-31G (d) here. The biggest difference between the calculated and the X-ray values of the bond length and bond angle of both **a** and **b** are at the 4-position of the pyridine ring. The calculated bond lengths are longer (0.1331 Å in **a**, C8-C9, and 0.127 Å in **b**, C1-C5) than the experimental values. The calculated bond angles are smaller (6.0508° in **a**, C8-C9-C8A, and 6.6361° in **b**, C5–C1–C2) than the X-ray values. The reason may be the inference by the intramolecular C-H···O H-bonds in both molecules.

### Vibration assignments

**Figure 5:**
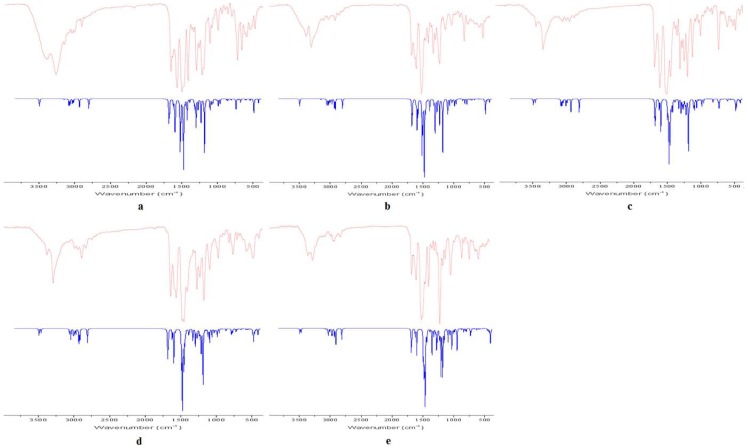
Experimental (Red) and simulated (Blue) Infrared spectra of a-e.

The FT-IR spectrum of the five compounds were recorded in the frequency region of 4000–400 cm^−1^, and the harmonic vibrational frequencies calculated by using B3LYP with 6-31G (d) basis set are given along with the experimental ones in **Table 4**. The FT-IR and predicted spectra for the compounds are given in **Fig. 5**. None of the predicted vibrational spectra have any imaginary frequency prove that optimized geometry is located at the lowest point on the potential energy surface. It is well known that DFT levels systematically overestimate the vibrational wave-numbers. So, the scaling factor values of 0.96 were used in order to correct anharmonicity and neglected part of electron correlation [12], [13]. The assignments of various bands in different compounds, in general, have been reported in detail [14], [15]. The B3LYP method with 6-31G (d) basis set has good ability to predict the IR spectra of the five compounds.

**Table 4:** Experimental and calculated vibrational frequencies (cm^−1^) with DFT method.

Assignments	a		b		c		d		e	
	Exp. (IR)	B3LYP/6-31G (d)	Exp. (IR)	B3LYP/6-31G (d)	Exp. (IR)	B3LYP/6-31G (d)	Exp. (IR)	B3LYP/6-31G (d)	Exp. (IR)	B3LYP/6-31G (d)
*ν*_N-H_	3405		3395		3407		3401		3373	
	3279	3282	3313		3306		3312		3294	
	3171	3161								
*ν*_ = C-H_	3070	3056	3097	3093	3050	3055	3012	3038		
	3017		3024	3037	3020					
*ν*_C-H_	2910	2921	2920	2920	2970		2916	2928	2942	2931
			2856	2918	2922	2927	2850		2841	
			2793		2847					
*ν*_C = O_	1678	1684	1673	1675	1681	1689	1675	1670	1675	1666
	1651	1653								
*ν*_C = C_	1593	1587	1596	1579	1605		1598	1640	1601	1622
	1525	1536	1509	1513	1505	1502	1517	1517	1515	1517
	1498	1492			1450	1448	1482	1491		
	1432	1446					1445	1446		
*δ*_C-H_	1369	1369	1400		1355	1352			1397	1408
	1351	1352								
*ν*_C-N_	1310	1325	1318	1325	1313	1312	1311	1320	1305	1288
	1290	1284	1285	1285	1283	1281	1268	1269	1214	1219
	1238	1231	1216	1217	1250	1267	1212	1236	1165	1183
	1210	1212	1121	1114	1205		1134	1142	1126	1139
	1118	1119	1014	1018	1132	1141	1012	1017	1036	1038
	1035	1030			1014	1021				
*γ*_ = C-H_	751	756	813	820	841	852	861		857	846
	692	694	751	755	751	753	808	807	822	830
*ν*_C-Cl_									732	718

### Frontier molecular orbital and energy

HOMO and LUMO energies are very important parameters for quantum chemistry. LUMO as an electron acceptor represents the ability to obtain an electron, whereas HOMO represents the ability to donate an electron [16]. Energy gap (Eg) between HOMO and LUMO characterizes the molecular chemical stability and it is a critical parameter in determining molecular electrical transport properties because it is a measure of electron conductivity [17].

**Fig. 4** shows the patterns of the HOMO and LUMO of the five compounds calculated with the B3LYP level. The positive phase is symbolized with blue and the negative phase green. HOMO and LUMO energies of the compounds are listed in **Table 5**.

**Table 5:** Frontier orbitals and energy gaps (Eg).

Comp.	HOMO-3	HOMO-2	HOMO-1	HOMO	LUMO	LUMO+1	LUMO+2	LUMO+3	Eg (eV)
**a**	−0.247	−0.220	−0.216	−0.196	−0.050	−0.011	0.000	0.000	0.146
**b**	−0.246	−0.215	−0.211	−0.195	−0.049	−0.010	0.000	0.000	0.146
**c**	−0.240	−0.225	−0.223	−0.194	−0.044	−0.005	0.003	0.004	0.15
**d**	−0.239	−0.220	−0.218	−0.193	−0.043	−0.003	0.003	0.004	0.15
**e**	−0.239	−0.215	−0.214	−0.192	−0.044	−0.018	−0.011	0.002	0.148

It can be seen that the Egs of all the five compounds are small (about 0.15 eV). They have delocalized π systems. It is easier for the vertical transitions of the delocalized π electrons from HOMO to LUMO.

### Molecular electrostatic potential map

The molecular electrostatic potential (MEP) map is useful to study the electrophile attracted negative regions (where the electron distribution effect is dominant) [18]. The importance of MEP lies in the fact that it simultaneously displays molecular size, shape as well as positive, negative and neutral electrostatic potential regions in terms of color grading. Regions of negative are usually associated with the lone pair of electronegative atoms.

**Figure 6:**
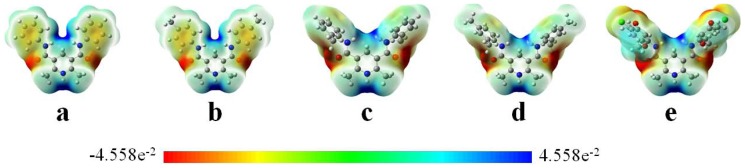
Molecular electrostatic potential of a–e.

**Fig. 6** shows the MEP map of the five molecules, where potential increases in the order of red < orange < yellow < green < blue. The regions having the negative potential are electron excess with the electronegative atoms (C = C group, oxygen and nitrogen atoms), while the regions having the positive potential are electron deficiency with hydrogen atoms.

### Electronic spectra

**Figure 7:**
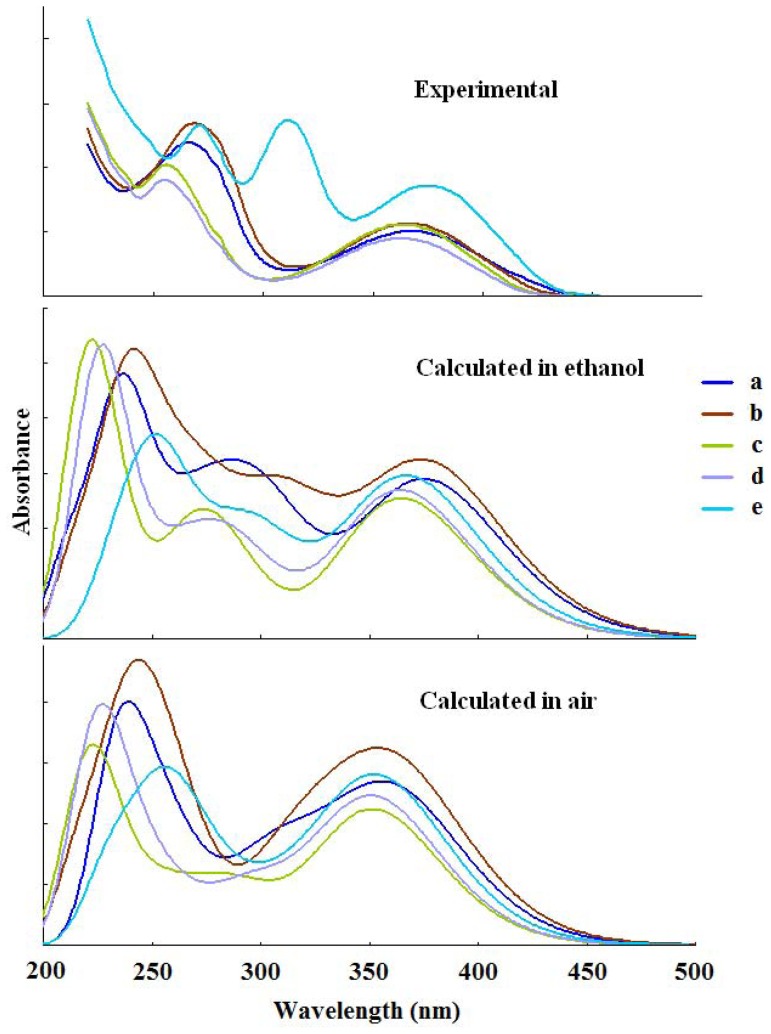
Experimental and calculated UV-vis spectra of a–e.

The electronic absorption spectra of **a–e** determined in ethanol are shown in **Fig. 7** and listed in **Table 6**. Since the presence of an aromatic ring and a heterocyclic, compounds of **a–e** have 2 to 3 absorption bands with the λ_max_ between 250 to 380 nm. Comparing to **a**, the absorption bands of **b–d** with alkyl substituent on the benzene rings are blue-shift. The absorption band of **e** with auxochrome groups of -OCH_3_ and -Cl is a certain degree of red-shift and the ε_max_ is also increased.

**Table 6:** Electronic absorption spectra of a–e in ethanol.

Comp.	λ_max_/nm	ε _λmax_/(L/mol·cm)
**a**	266/368	2.39×10^4^/1.01×10^4^
**b**	268/366	2.66×10^4^/1.10×10^4^
**c**	256/364	2.04×10^4^/1.06×10^4^
**d**	254/364	1.81×10^4^/0.88×10^4^
**e**	272/310/373	2.61×10^4^/2.74×10^4^/1.72×10^4^

To further understand the electronic transitions of **a–e**, TDDFT/B3LYP/6-31G (d) was used to study the nature and the energy of absorption spectra on the basis of the optimized geometries. The electronic absorption spectra were simulated by the Gaussian functions based on the 20 lowest singlet energies from the calculations and illustrated in **Fig. 7**. **Table 7** lists the main transition configurations and oscillator strengths for the most relevant singlet excited states of **a–e** both in vacuum and in ethanol.

**Table 7:** Calculated absorption spectra of a–e in vacuum and in ethanol.

Molecular	States	Transition	Coefficient	Strength *f*	λ (nm) (cal.)	λ (nm) (exp.)	Relative error (%)
**a**	gas-phase	S_0_→S_1_	0.66371	0.3068	361		1.9
	ethanol		0.66600	0.3530	376	368	−2.1
**b**	gas-phase	S_0_→S_1_	0.66471	0.3433	363		0.8
	ethanol		0.66743	0.3852	377	366	−2.9
**c**	gas-phase	S_0_→S_1_	0.65822	0.2732	352		3.4
	ethanol		0.66113	0.3138	365	364	−0.3
**d**	gas-phase	S_0_→S_1_	0.65954	0.2948	353		3.1
	ethanol		0.66192	0.3315	365	364	−0.3
**e**	gas-phase	S_0_→S_1_	0.66073	0.3270	355		5.1
	ethanol		0.66218	0.3605	368	373	1.4

The data of calculation and experiment are basically identical. The relative errors calculated in ethanol and in gas phase are 0.3–2.9% and 0.8–5.1%, respectively. Compared with the experimental data, calculated values in solution are superior to that in gas phase. The model considering the effect of the solvent is closer to the actual situation than the model of gas phase.

### Fluorescence spectra

The fluorescence mechanism can be simply expressed as: S_1_→S_0_+hν. The electrons from the first excited state drop back to the ground state and emit the radiation of degradation. Strong fluorescent substances have such structure characteristics that the molecules have rigid plane, greater delocalization of π bond, and lower singlet electronic excited states. Geometry optimization results show that the five compounds have the above characteristics of configuration.

**Figure 8:**
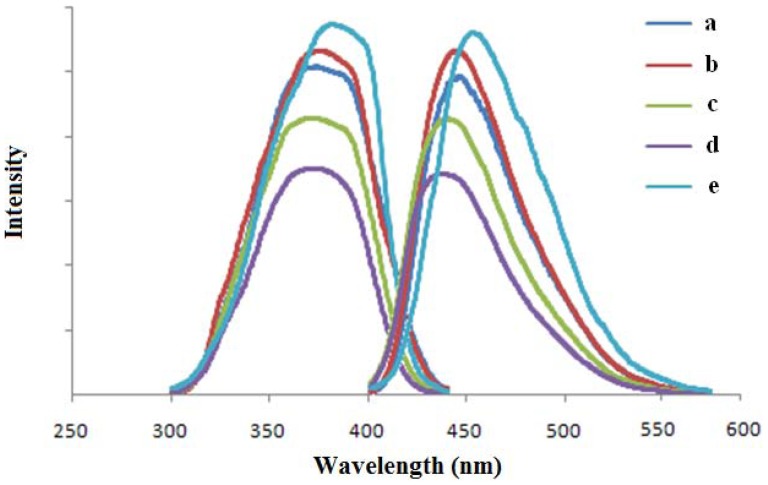
Experimental fluorescence spectra of a–e.

The fluorescence spectra of **a–e** determined in ethanol are shown in **Fig. 8** and listed in **Table 8**. The excitation and emission spectra of **a–e** are similar. The maximum excitation and emission wavelengths are near 375 nm and 450 nm, respectively. The Stokes shift is about 71–80 nm. The results indicate that the alkyl substitution has the effect of blue-shift on the fluorescence spectra of **b–d**. The maximum excitation and emission wavelengths of **e** with auxochrome groups of -OCH_3_ and -Cl are a certain degree of red-shift and the Stokes shift is bigger because the planarity of the molecule structure is increased by the lone pair electrons of -OCH_3_ and -Cl conjugating with the benzene rings.

**Table 8:** Florescence spectra of a–e in ethanol.

Comp.	λ_ex_ (nm)	λ_em_ (nm)	Stokes shift (nm)
**a**	374	452	78
**b**	375	450	75
**c**	370	444	74
**d**	370	441	71
**e**	380	460	80

## Conclusions

Five new 1,4-dihydro-2,6-dimethyl-3,5-pyridinedicarboxamides **a–e** have been synthesized and characterized by spectrometry and X-ray diffraction.

The experimental electronic absorption spectra in ethanol solution show 2 to 3 absorption bands with the λ_max_ between 250 to 380 nm. The predicted electronic absorption spectra were achieved by TDDFT in gas phase and in ethanol solution. The model considering the effect of the solvent is closer to the actual situation than the model of gas phase.

The results of the single crystal X-ray show that the compounds look like beautiful butterflies. The same intramolecular C-H···O H-bonds in the molecules result in the formation of four non-planar pseudo rings with envelope conformations. The geometric parameters calculated by DFT/B3LYP/6-31G (d) represent a good approximation to the experimental data.

The present work will be helpful to systematically understanding of the structures and the optical properties of 1,4-dihydropyridines for studying the structure-activity relationship and to develop new drugs and their analytical methods.

## Experimental

### Materials and instruments

Acetoacetanilides (Sinopharm Chemical Reagent Ltd.). Other chemicals and solvents were reagent grade and were used without further purification.

Melting points were measured on an X-4 microscope electrothermal apparatus (Taike, China) and were uncorrected. IR spectra were obtained on a Nicolet 380 FT-IR spectrophotometer (KBr Pellets). ^1^H NMR spectra were recorded on a Bruker AV-300 spectrometer at 300 MHz using CDCl_3_/DMSO-*d6* as the solvent, with tetramethylsilane (TMS) as internal standard. The chemical shifts were reported in δ ppm and the coupling constants in J Hz. The elemental analyses were performed with a Flash EA-1112 elemental analyzer. Electronic absorption spectra were obtained using a Cary5000 UV/vis/near-IR Spectrophotometer (Varian). The X-ray crystallographic analysis was performed on a Nonius CAD4 single-crystal diffractometer using graphite-monochromated Mo Kα radiation (λ = 0.71073) Å. Purity of the compounds was checked on thin layer chromatography (TLC) plates (silica gel G), the spots were located under UV light. Fluorimetric measurements were carried out using a spectrofluorometer (FP-6200, Jasco) equipped with a xenon lamp, dual monochrometers, and a controlling computer with operating software (Microsoft Windows). The slit widths for both excitation and emission were set at 5 nm. The sample solution was transferred to a conventional 1×1-cm quartz cell and then mounted on a cell holder. Subsequently, fluorescence spectra and their associated intensities were observed using the standard method.

Yellow color crystals of **a** and **b** suitable for X-ray analysis were grown from ethanol. A crystal was put on a glass fiber. The diffraction data were collected by using a *x/2h* scan mode at 293 K. The crystal structure was solved by the direct method and refined by the full-matrix least-squares procedure on *F* ^2^ using SHELXL-97 program [19]. Positions of hydrogen atoms were located by geometrical calculation (*x, y, z* and *U*_iso_ fixed to 1.2 times *U*_iso_ of the atom they are bound to).

### Computational details

DFT methods of hybrid B3LYP were used to optimize the molecular structures and to study the properties of **a–e**. The 6-31G (d) and 6-31G (d, p) basis sets were used. The electronic absorption spectra were calculated and simulated with the time dependent density functional theory (TDDFT) method. All calculations were carried out using the Gaussian 09 program [20].

### General procedure for synthesis of a–e

A mixture of acetoacetanilide derivative (0.01 mol), hexamethylene tetramine (0.01 mol), ammonium acetate (0.005 mol), and water (5 mL) were transferred to a round bottom flask containing 15 mL of ethanol. The reaction mixture was refluxed for 10–16 h. The reaction was monitored by TLC using the solvent system (ethyl acetate: petroleum ether  = 2∶1). Soon after the reaction was completed, the reaction mixture was allowed to cool. The solid product formed was filtered and washed with cold ethanol to get the 1,4-DHPs. The physical properties and ^1^H NMR data of **a–e** are listed in **Table 9** and **Table 10**, respectively.

**Table 9:** Yield, melt point, and EA data of compounds a–e.

Compd.	Yield (%)	Physical state	m.p./°C	Elemental anal. (%, Calcd.)
**a**	78.3	Light yellow cryst.	224–226	C 72.36(72.60), H 6.11(6.09), N 12.15(12.10)
**b**	76.1	Yellow cryst.	232–234	C 73.32(73.57), H 6.74(6.71), N 11.15(11.19)
**c**	70.3	Light yellow cryst.	244–247	C 73.31(73.57), H 6.68(6.71), N 11.23(11.19)
**d**	73.8	Light yellow cryst.	307–309	C 74.69(74.41), H 7.27(7.24), N 10.36(10.41)
**e**	62.6	Yellow cryst.	238–241	C 56.23(55.98), H 5.10(5.07), N 7.79(7.83)

**Table 10:** ^1^H NMR data °f compounds a–e.

**a**	9.06 (s, 2 H, NH), 7.76 (s, 1 H, NH), 7.64–6.98 (m, 10 H, Ar-H), 3.41 (s, 2 H, CH_2_), 2.02 (s, 6 H, CH_3_)
**b**	8.95 (s, 2 H, NH), 7.71 (s, 1 H, NH), 7.50 (d, J = 8.6 Hz, 4 H, Ar-H), 7.07 (d, J = 8.6 Hz, 4 H, Ar-H), 3.38 (s, 2 H, CH_2_), 2.24 (s, 6 H, CH_3_), 2.01 (s, 6 H, CH_3_)
**c**	8.49 (s, 2 H, NH), 7.77 (s, 1 H, NH), 7.36–7.04 (m, 8 H, Ar-H), 3.49 (s, 2 H, CH_2_), 2.20 (s, 6 H, CH_3_), 2.09 (s, 6 H, CH_3_)
**d**	8.42 (s, 2 H, NH), 7.72 (s, 1 H, NH), 7.20–6.93 (m, 6 H, Ar-H), 3.45 (s, 2 H, CH_2_), 2.25 (s, 6 H, CH_3_), 2.15 (s, 6 H, CH_3_), 2.08 (s, 6 H, CH_3_)
**e**	8.34 (s, 2 H, NH), 7.84 (s, 1 H, NH), 6.93 (d, J = 4.8 Hz, 2 H, Ar-H), 6.91 (d, J = 1.7 Hz, 2 H, Ar-H), 3.94 (s, 6 H, OCH_3_), 3.85 (s, 6 H, OCH_3_), 3.51 (s, 2 H, CH_2_), 2.25 (s, 6 H, CH_3_)

## Supplementary material

Crystallographic data for the structural analysis of the synthesized compounds have been deposited at the Cambridge Crystallographic Data Center, 12 Union Road, Cambridge, CB2 1EZ, UK, and are available free of charge from the Director on request quoting the deposition number CCDC 851885 and 851886 (fax: C44 1223 336033, e-mail: deposit@ccdc.cam.ac.uk).
